# Atypical Vascular Proliferation Secondary to Radiotherapy in a Patient With a History of Synovial Sarcoma

**DOI:** 10.7759/cureus.10179

**Published:** 2020-09-01

**Authors:** Carlos E Bonilla, Lucy M Perez Lugo, Camilo Vallejo Yepes, Handerson R Osma Charris

**Affiliations:** 1 Oncology, Instituto Nacional De Cancerología, Bogota, COL; 2 Internal Medicine, Instituto Nacional De Cancerología, Bogota, COL

**Keywords:** angiosarcoma, atypical vascular proliferation, c-myc, synovial sarcoma, angiogenesis, cutaneous vascular neoplasms

## Abstract

We present the case of a 21-year-old male patient with a history of monophasic synovial sarcoma in his left thigh, which was treated with surgical resection, radiotherapy, and chemotherapy with mesna, doxorubicin, and Ifosfamide (MAI protocol). Approximately six years after the end of the oncological treatment, he presented a nodular, polypoid lesion in the left popliteal region, which was painless and fast growing. Ultimately, the biopsy was consistent with atypical vascular proliferation (AVP). Vascular lesions after radiotherapy include a wide spectrum of pathologies that range from benign lesions such as AVP to malignant ones with very poor prognosis such as angiosarcoma, the distinction between one and the other can be difficult, being the determination rearrangement or amplification of gene c-myc, a key to make an accurate diagnosis in case of doubt.

## Introduction

An atypical vascular lesion is an unusual complication following treatment with radiation therapy for breast cancer and other diseases [1]. It covers a spectrum of diseases ranging from benign lesions such as atypical vascular proliferation (AVP) to truly malignant entities such as angiosarcoma [2]. Occasionally, it can be difficult to distinguish histologically between these entities so is necessary to rely on molecular studies such as the amplification of c-myc gene, this test can be positive in up to 71% of angiosarcoma cases [1-3]. We report the case of a patient with AVP following treatment with adjuvant radiation therapy for a left lower extremity synovial sarcoma, treated with surgical resection, subsequently without relapse episodes to date; determination of rearrangements or amplifications of the c-myc gene was vital to rule out angiosarcoma, identify prognosis and, therefore, there was no need for additional therapies.

## Case presentation

**Figure 1:**
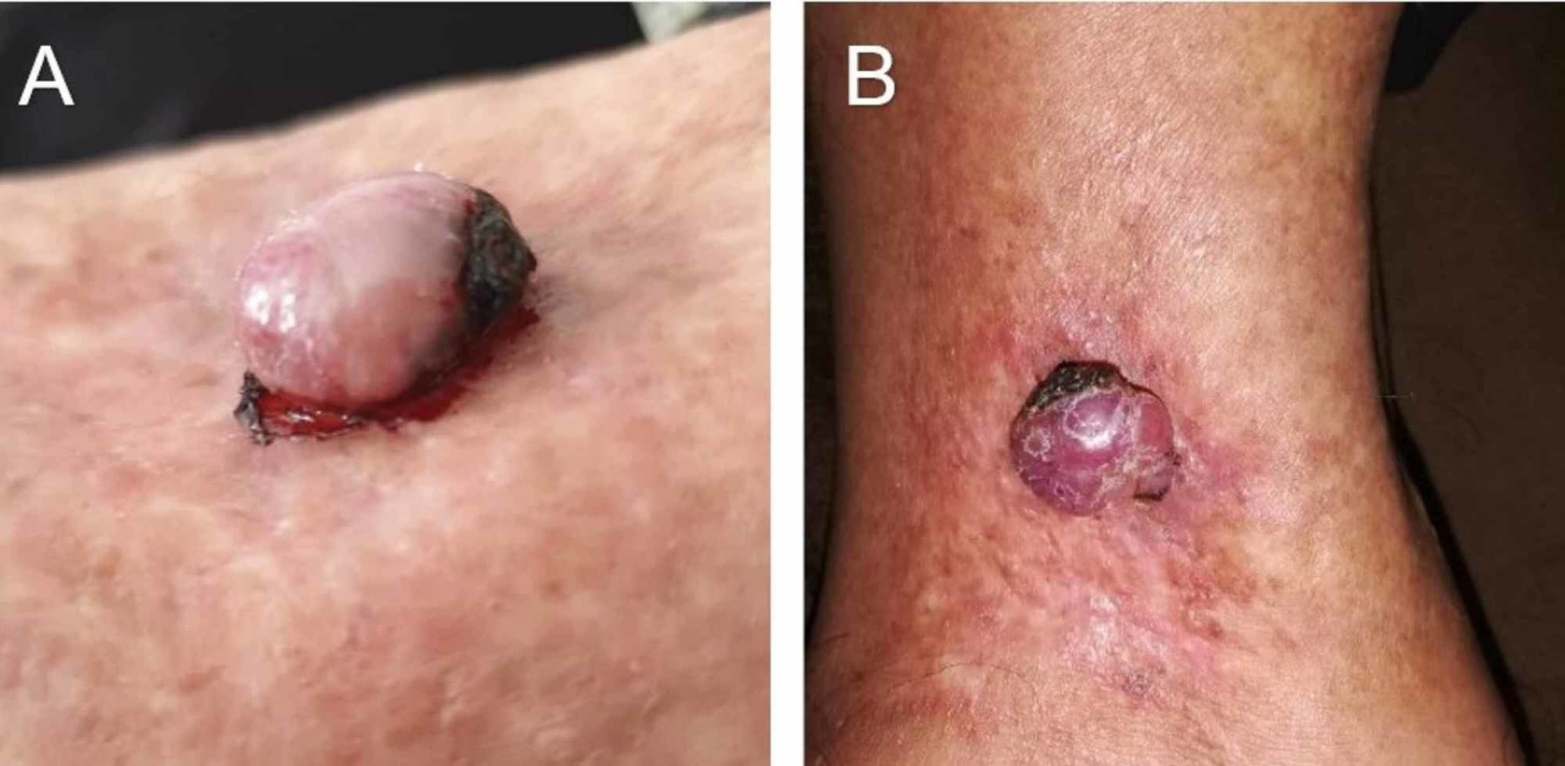
Nodular lesion A. Lateral view of patient’s multilobulated polypoid lesion with eccentric necrotic area that appeared six years after radiotherapy. B. Superior view of the same lesion.

**Figure 2:**
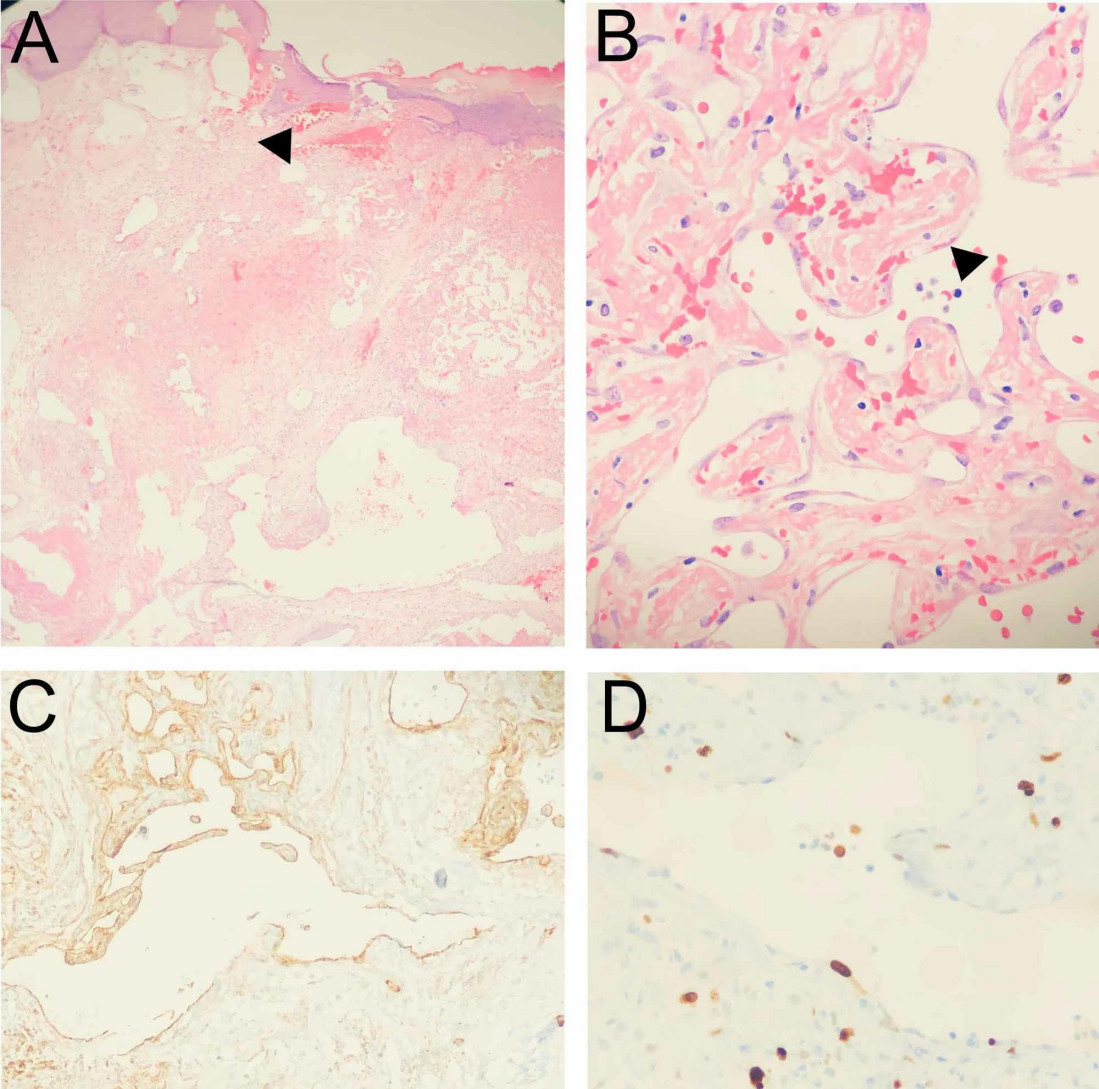
Histopathological assessment A: Skin with acanthosis and atypical vascular proliferation at dermis level made up of dilated, thin-walled congestive vessels with reactive-looking endothelium is observed in the hematoxylin-eosin (HE)–stained cuts (black arrow). B: There are also vascular areas constituted by papillary projections with hyalinized centers and focal areas of stroma of necrotic aspect (black arrow). C: This vascular proliferation shows immunoreactivity for the immunohistochemical markers, cluster of differentiation 31 (CD31) and FLI1 (in a focal way). D: There is negativity for the latent nuclear antigen-1 (LNA1) and human herpesvirus 8 (HHV8). Little cell proliferation is observed when measured with Ki67.

A 21-year-old male patient with a history of stage III monophasic synovial sarcoma in the left thigh (pT2bN0M0) in 2012 was treated with surgical resection, followed by adjuvant radiotherapy with 6600 cGY in 33 fractions and four sequential chemotherapy cycles with mesna, doxorubicin and ifosfamide (MAI protocol). In 2018, he presented a painless polypoid lesion in the left popliteal fossa region with rapid growth, reaching 1.5 cm in diameter (Figure 1). An excisional biopsy was performed and histopathological analysis reported a vascular proliferation with immunohistochemistry positive for cluster of differentiation 31 (CD31), negative for human herpesvirus 8 (HHV8), 20% Ki67 and focal areas of stroma of necrotic aspect, suggesting a diagnosis of AVP versus angiosarcoma (Figure 2). Due to the diagnostic uncertainty, fluorescence in situ hybridization (FISH) of the c-myc gene was performed with negative results, confirming the diagnosis of AVP and reasonably ruling out the possibility of secondary angiosarcoma. At the clinical follow-up in 2020, after two years of resection of the vascular lesion, the patient remains free of relapses.

## Discussion

AVP is a rare complication after treatment with radiotherapy [4], usually described after treatment of breast and other gynecological cancers [4,5], and less frequently in other types of neoplasms [6-9]. This complication is included in a spectrum of vascular entities in previously treated sites that includes benign lesions, atypical proliferation, and finally angiosarcoma [2,10,11]. They usually occur three-to-six years after treatment with radiation therapy [5,12]; however, there are reports of cases as early as eight months [13]. Benign lymphangiomatous lesions usually present as well-circumscribed, solitary papules that in histopathology show no atypia or proliferative activity. In AVP, the usual clinical presentation includes the presence of papules with coloring ranging from red to brown, most of them smaller than 20 mm [14,15]. The appearance of up to four synchronous lesions has been reported, however, the most common form of presentation is the isolated presence of a single lesion as described by Brenn et al. in a series of 42 cases [16].

The term atypical vascular lesion or proliferation was introduced by Rosen and Fineberg in 1994 in a series of cases in which they described the similarities and differences with angiosarcoma [17,18]. AVP usually have a benign course, without fatal complications or secondary metastatic staining [14]. It is described more frequently in women, probably because the pathology in which it has been found most often is posterior to the treatment of breast cancer. In the review of 193 cases of vascular proliferation by Zhong et al., the condition in men corresponded to only 1.6% of cases [6].

Since it shares some similarities with secondary angiosarcoma histologically [1] but with a different prognosis and management approach, it is important to make a diagnosis with certainty. Table 1 describes some of the histopathological characteristics that allow this distinction to be made.

**Table 1:** Histopathological characteristics that differentiate angiosarcoma from atypical vascular proliferation [17,18].

Histopathological characteristic	Atypical vascular proliferation	Angiosarcoma
Subcutaneous infiltration	-	+++
Papillary endothelial hyperplasia	-	+++
Protruding nucleoli	-	+++
Mitotic figures	-	+++
Significant cytological atypia	-	+++
Blood lakes	-	++
Dermal collagen dissection	±	+++
Anastomotic cups	++	+++
Hyperchromic endothelial cells	+++	++
Chronic inflammation	+++	+
Relatively circumscribed	+++	-
Projection of the stroma to the lumen	+++	-
Cluster of differentiation (CD)34	++	+
CD31	+++	+++
D2-40	++	+
MYC expression	-	+++
P53	++	++
Prox1	-/+	+++

Although some histopathologic features may help differentiate AVP from angiosarcoma, they may have common characteristics that make diagnosis difficult. The myc genes are a family of proto-oncogenes including c-myc, L-myc, and N-myc, with c-myc being the most widely used. This gene has three exons located on chromosome 8q24 which plays an important role in cell division, growth and apoptosis [19]. FISH analysis for c-myc rearrangement is used to distinguish atypical vascular lesions from angiosarcoma, with positive rearrangement or amplification in cases of radiation-supported angiosarcoma and negative in atypical vascular lesions [1-3].

The clinical behavior of the AVP is benign, and to date no cases of progression or death associated with the condition have been reported. However, new lesions in the same field of irradiation with potential expression of c-myc and progression to angiosarcoma may occur during follow-up [16]. The treatment of choice is complete surgical resection of the lesion, and if new lesions appear in the radiation field, a new biopsy should be performed [20].

## Conclusions

AVP is a rare post-radiotherapy skin complication, usually described after breast cancer treatment and only rarely in other pathologies. The histopathological study of vascular lesion after radiotherapy is important, because the spectrum of pathologies includes frankly benign entities, AVP, and finally angiosarcoma, which differ in prognosis and treatment. The use of immunofluorescence techniques to evaluate c-myc rearrangement or amplification is useful in cases with diagnostic doubt.
